# Ghrelin promotes neurologic recovery and neurogenesis in the chronic phase after experimental stroke

**DOI:** 10.1186/s42466-025-00371-6

**Published:** 2025-03-03

**Authors:** Carolin Beuker, Ulrike Schreiner, Jan-Kolja Strecker, Elena Altach, Verena Rätzel, Antje Schmidt-Pogoda, Heinz Wiendl, Jens Minnerup, Kai Diederich

**Affiliations:** 1 Department of Neurology with Institute of Translational Neurology, Medical Faculty, University Hospital Muenster, 48149 Muenster, Germany; 2 Clinic of Neurology and Neurophysiology, Medical Center, Faculty of Medicine, University of Freiburg, 79106 Freiburg, Germany; 3 Department of Neurology, University of Lübeck and University Medical Center of Schleswig- Holstein, Campus Lübeck, Ratzeburger Allee 160, 23538 Lübeck, Germany

**Keywords:** Ghrelin, Neurogenesis, Regeneration, Stroke

## Abstract

**Background:**

The neuroprotective and proangiogenic potential of ghrelin in acute ischemic stroke has been demonstrated in experimental studies. However, the transferability of these results is limited as ghrelin was administered either before or very early after stroke onset and follow-up was limited to the first days after stroke. The aim of this study was therefore to close and extend this knowledge gap. To this end, we investigated the effect of ghrelin in two different translational animal models, one investigating acute and one investigating long-term structural and functional recovery after experimental stroke.

**Methods:**

Middle cerebral artery occlusion (MCAO) or photothrombotic stroke was induced in 65 adult male Wistar rats. Eleven sham-operated animals served as controls. The rats were treated with either ghrelin, the ghrelin receptor antagonist [D-Lys]-GHRP-6 or a control substance. Up to four weeks after ischemia, behavioral tests such as the cylinder test, the tape removal test, and the rotarod test were performed to examine sensorimotor deficits, and the Morris water maze was performed to examine effects on the acquisition and consolidation of new memories. The structural outcome was determined by a differential analysis of neurogenesis in relation to survival and proliferation of newborn neurons in the post-ischemic brain, angiogenesis and determination of infarct size.

**Results:**

Ghrelin treatment improved motor and somatosensory functions and preserved the consolidation of new memories after photothrombotic stroke. As a structural correlate, long-term survival and sustained proliferation of neuronal cells after stroke was significantly increased in ghrelin-treated rats, while angiogenesis remained unaffected. In contrast to these neuroregenerative mechanisms, ghrelin did not induce immediate neuroprotective effects after MCAO.

**Conclusions:**

Our results suggest that ghrelin has a significant pro-neuroregenerative effect by enhancing long-term survival and sustained proliferation of neurons in the dentate gyrus and peri-infarct area, thus promoting functional recovery. Overall, ghrelin represents a promising target in the subacute and chronic phase after ischemic stroke.

**Supplementary Information:**

The online version contains supplementary material available at 10.1186/s42466-025-00371-6.

## Introduction

Despite tremendous research efforts, ischemic stroke remains one of the most common causes of disability worldwide [1] and recovery is generally far from complete. The observation that approximately one-third of patients who suffer a stroke develop some form of cognitive impairment [2] and 10–30% may develop dementia [3] underscores the enormous burden of the disease and the need for effective treatment options. Therefore, there is an urgent need for strategies to promote recovery in order to effectively support the body’s repair mechanisms after a stroke.

The contribution of neurogenesis in post stroke regeneration is potentially complex and, despite considerable research efforts in recent years, is not fully understood. Post-stroke neurogenesis is closely linked to angiogenesis, another regenerative process that occurs in response to cerebral ischemia [4–6]. This process is defined as the formation of new blood vessels through the sprouting of pre-existing vessels. Angiogenesis provides a favorable environment for neurogenesis, as growth factors and other signaling molecules released during angiogenesis can stimulate neural stem cells and promote the survival and differentiation of new neurons. Conversely, the presence of newly formed neurons can also release factors that support angiogenesis.

Due to its neuroprotective and neurogenic properties, ghrelin, a 28-amino acid peptide, is a promising agent for the treatment of ischemic stroke. The peptide is thought to have numerous functions beyond its prominent role in nutrition and metabolism [7, 8], including a number of important neural functions such as reward perception and motivation [7, 8] as well as learning and memory [9]. There is evidence from preclinical animal studies that ghrelin has a neuroprotective effect in various neurological diseases such as Parkinson’s disease or Alzheimer’s disease [10, 11], but also in ischemic stroke [12, 13]. In terms of mechanism of action, ghrelin has been shown to stimulate the proliferation, differentiation and migration of neural stem/progenitor cells (NS/PCs) [14, 15], which are known to be involved in repair mechanisms following ischemic stroke [16–20]. Ghrelin has also been shown to have beneficial effects on learning and memory formation, which may be directly related to its neurogenic properties [9, 21, 22].

However, the current evidence on the neuroprotective and neuroregenerative properties of ghrelin is partly contradictory and focuses on a limited period after stroke [23–26]. The aim of the present study was, first, to investigate the immediate neuroprotective effects of ghrelin on functional and structural outcome after transient middle cerebral artery occlusion in rats. Second, we investigated the role of ghrelin in neuroregeneration in the chronic phase after cerebral ischemia using the photothrombotic stroke model, which is characterized by the reliable evocation of neuroregenerative events such as neurogenesis and angiogenesis [27].

## Materials and methods

### Animals

Adult (12–13 weeks of age, *n* = 65) male Wistar rats were used for all experiments. Rats were housed in groups of four in Makrolon type IV cages (55.6 × 33.4 × 19.5 cm) equipped with bedding, nesting material and shelter and had ad libitum access to pelleted food and water. Rats were maintained under standard conditions at an ambient temperature of 20–24 °C and humidity of 45-65% on a 12:12 h light-dark cycle. Sham-operated Wistar rats served as controls.

### Sample size calculation

A detailed description of the calculation of the sample size can be found in the supplementary methods.

### Middle cerebral artery occlusion

A detailed description of the induction of middle cerebral artery occlusion can be found in the supplementary methods.

### Photothrombotic Stroke

A detailed description of the induction of photothrombotic stroke can be found in the supplementary methods.

### Experimental design

**Fig. 1:**
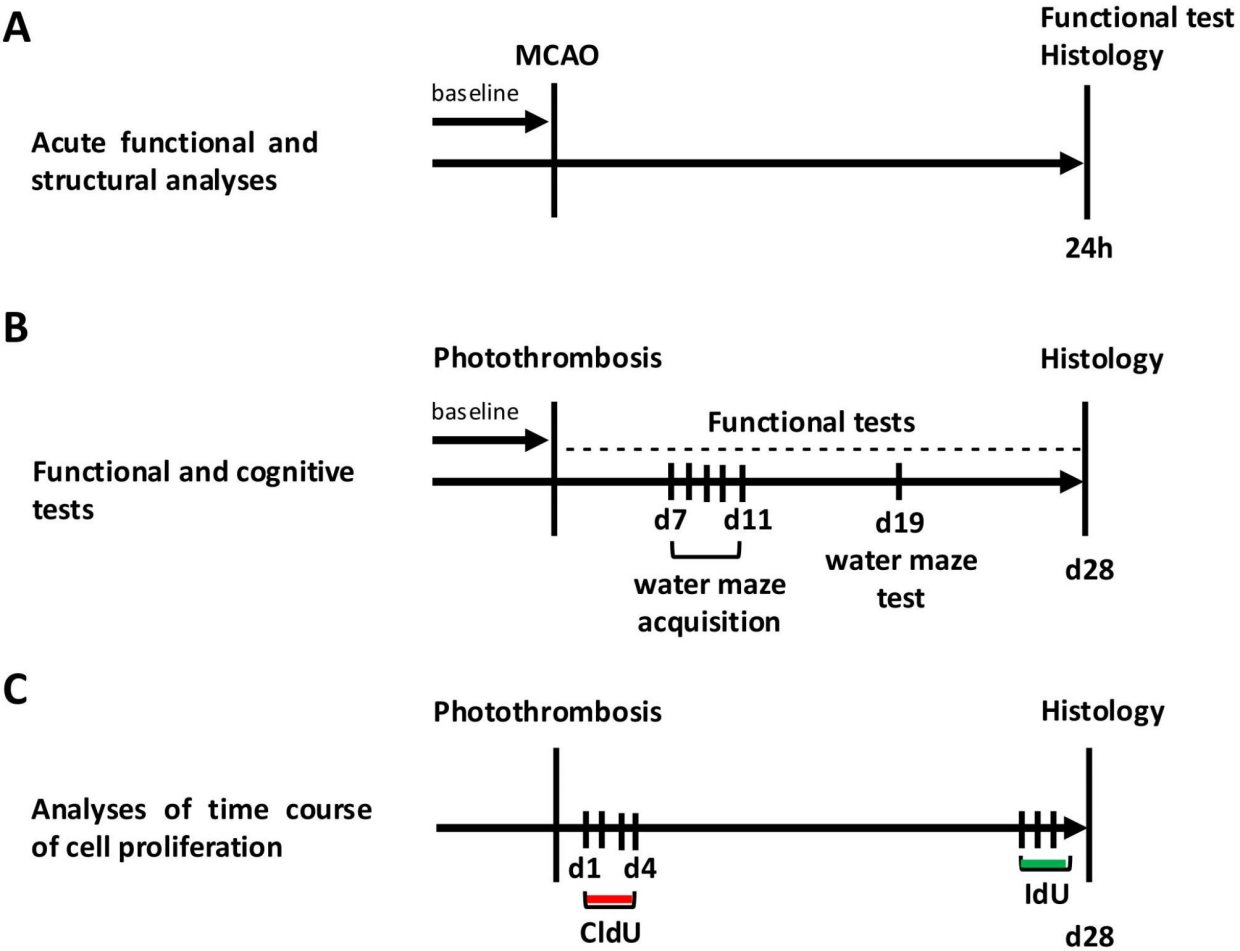
Experimental schedule. **(A)** Analysis of acute tissue injury (immunohistochemistry) and functional outcome after experimental stroke. Middle cerebral artery occlusion (MCAO) was induced in rats for 45 min, and rats were sacrificed 24 h later (after ischemia). **(B)** Behavioral tests to determine the effects of photothrombotic stroke on the acquisition of new memory content and sensorimotor functions. **(C)** Time course analyses of cell proliferation using CldU (chloro-deoxyuridine) and IdU (iodo-deoxyuridine) injections

Figure 1 shows a graphical summary of the individual experiments. A detailed description of the experimental design can be found in the supplementary methods.

### Behavioral assessment

A detailed description of the behavioral assessment can be found in the supplementary methods.

### Tissue collection and processing for histology

Twenty-four hours after MCAO or 28 days after photothrombosis, rats were perfused through the left ventricle with phosphate buffered saline (PBS) for 5 min followed by 4% paraformaldehyde solution for 10 min under deep xylazine/ketamine anesthesia. Brains were removed, fixed in 4% paraformaldehyde overnight, immersed in 30% sucrose for three days, frozen and stored at -80˚C.

### Infarct volume assessment

A detailed description of the infarct volume assessment can be found in the supplementary methods.

### Immunohistochemistry following photothrombosis (experiment 2)

A detailed description of the processing of the brains for immunohistochemistry can be found in the supplementary methods.

### Assessment of neuroregenerative mechanisms

A detailed description of the assessment of neuroregenerative mechanisms can be found in the supplementary methods.

### Randomization and blinded assessment

Allocation to the experimental groups and selection for outcome assessment was randomized using the Research Randomizer [28]. All caregivers and investigators were blinded for the intervention and all outcomes were assessed in a blinded manner by a single experienced medical technical assistant and reviewed by KD.

### Statistical analysis

Statistical analysis was performed using R version 4.1.1 and GraphPad Prism version 8 (GraphPad Software, La Jolla, CA). Data were tested for normal distribution using the Shapiro-Wilk normality test. Behavioral tests were analyzed by 2-way repeated measures ANOVA. One-way ANOVA followed by Fisher’s protected least significant difference (LSD) post hoc test was used for comparisons with > 2 groups. Student t test was used for comparisons between 2 groups. If a normal distribution was not given, the Kruskal-Wallis test and Dunn’s post hoc test were applied. Data are presented as mean ± SD. A p value of < 0.05 was considered significant.

## Results

### Ghrelin does not affect early neurological outcomes after MCAO

**Fig. 2:**
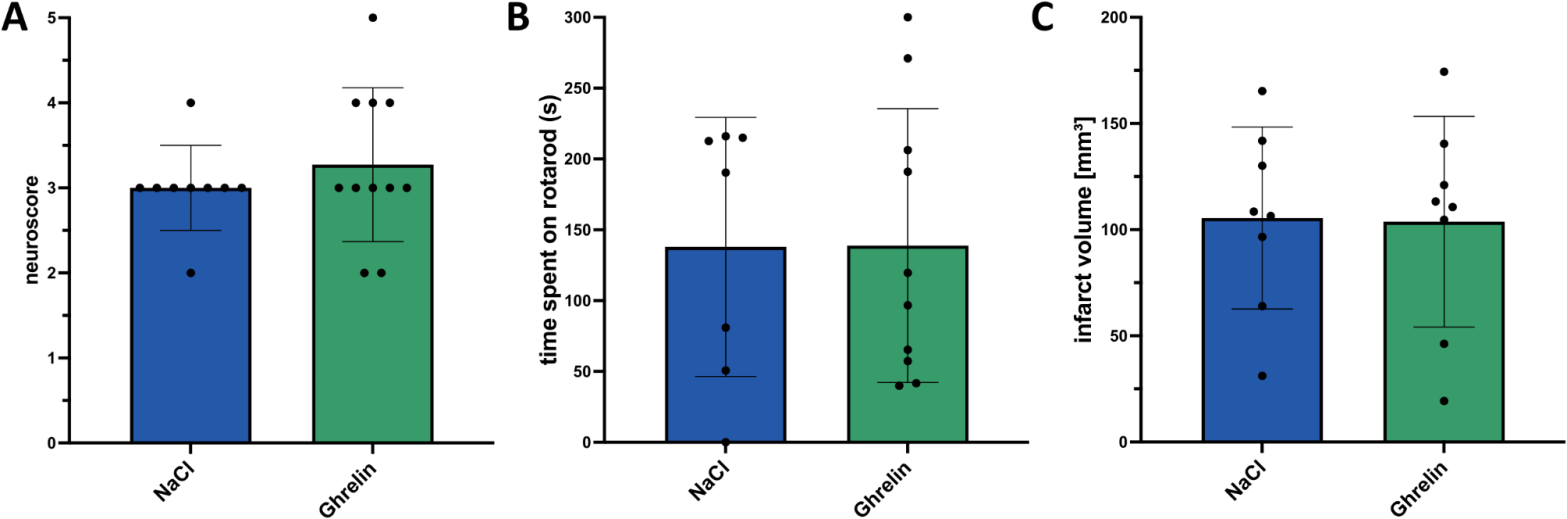
Analysis of early functional and structural outcome. **(A)** Neuroscore and **(B)** rotarod performance 24 h after MCAO. **(C)** Mean infarct volumes were calculated from coronal cryosections (15–20) taken at 240 μm intervals 24 h after MCAO and stained with toluidine blue using ImageJ software. Two-sided *t* test

Since ghrelin has recently been shown to have neuroprotective properties, we wanted to investigate whether ghrelin administration improves early structural and functional outcomes. Neuroscores and rotarod performance were not significantly different between the ghrelin group and vehicle-treated animals (*t* test, *p* = 0.43 and *p* = 0.98; Fig. 2A, B). Infarct volumes 24 h after MCAO did not differ significantly between rats treated with ghrelin or vehicle (*t* test, *p* = 0.94; Fig. 2C). Our results indicate that ghrelin has no effect on early functional and structural outcome after ischemic stroke.

### Ghrelin enhances sensorimotor long-term recovery after photothrombotic stroke

**Fig. 3:**
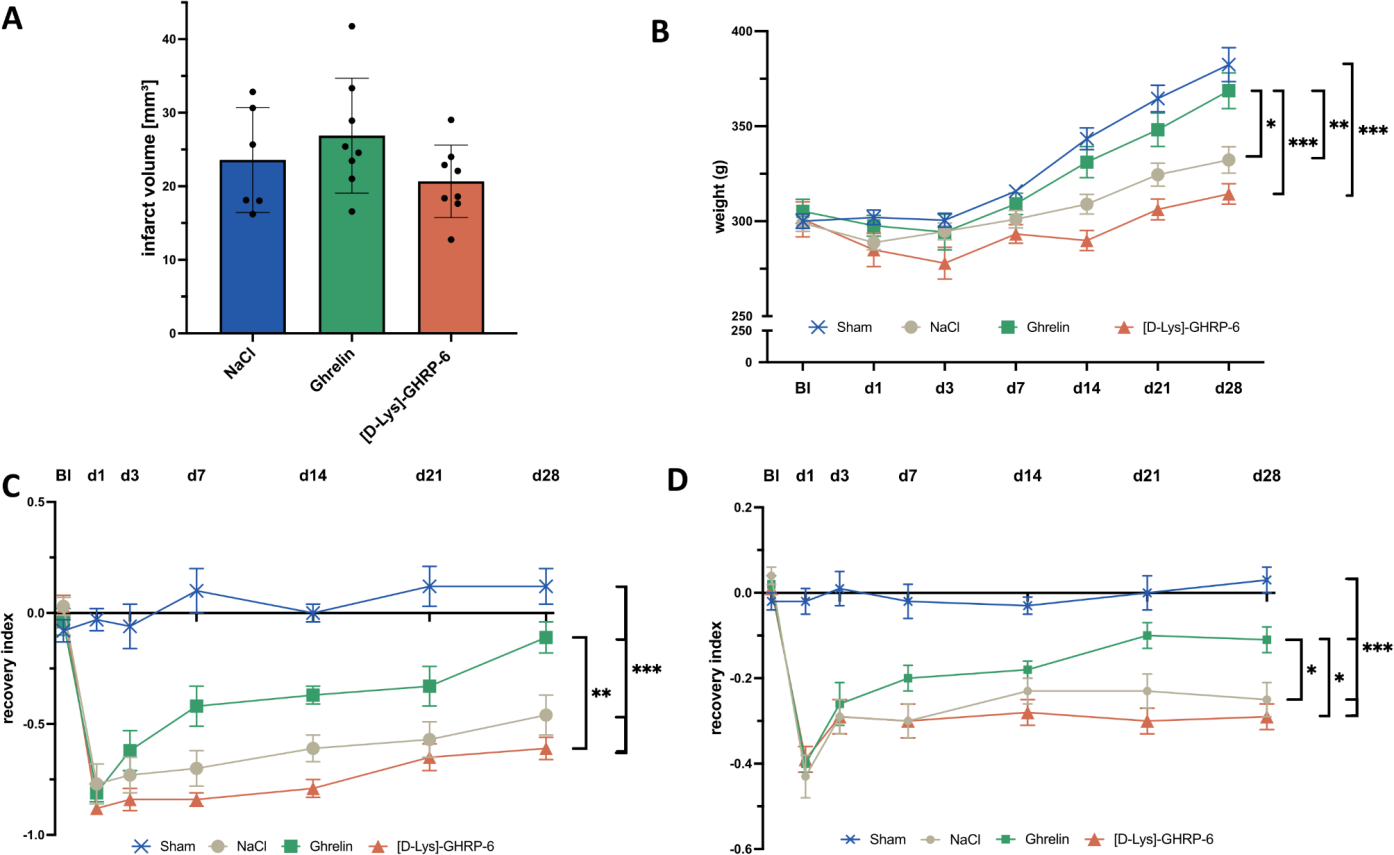
Evaluation of long-term structural and functional outcomes. **(A)** Mean infarct volumes calculated 28 days after photothrombotic stroke. **(B)** Weight progression. **(C)** Somatosensory recovery as assessed by the adhesive tape removal test. **(D)** Motor deficits as assessed by the cylinder test. ANOVA with repeated measures and one-way ANOVA followed by Fisher’s LSD; *P < 0.05, **P < 0.01, ***P < 0.001

Next, we examined the role of ghrelin on neuroregeneration in the chronic phase after cerebral ischemia. Infarct volumes 28 days after photothrombotic stroke did not differ significantly between rats treated with ghrelin or the ghrelin specific receptor antagonist and vehicle-treated rats (one-way ANOVA, *p* > 0.05 for all comparisons; Fig. 3A**)**. Second, we investigated the possible long-term effects of ghrelin on body weight because it is known to stimulate appetite, food intake, and weight gain. Therefore, the weight of the experimental animals was recorded throughout the experimental studies. During the postischemic phase, animals in all groups gained weight. Ghrelin-treated animals gained significantly more weight compared to animals in the control group (repeated-measures ANOVA, *p* = 0.03; Fig. 3B). There was also significant weight gain in ghrelin-treated animals compared to antagonist (repeated-measures ANOVA, *p* = 0.001; Fig. 3B) and sham-operated animals compared to antagonist (repeated-measures ANOVA, *p* < 0.001; Fig. 3B).

The adhesive tape removal test was used to examine the somatosensory recovery. Motor recovery was assessed using the cylinder test. Baseline performance was comparable between all treatment groups. As expected, animals in all experimental groups showed marked deficits in somatosensory and motor function after photothrombotic stroke, which attenuated over 28 days until the end of the experiment (Fig. 3C, D). Analysis of the adhesive tape removal test revealed significant improvement in somatosensory scores in rats treated with ghrelin compared to rats treated with the antagonist (repeated-measures ANOVA, *p* = 0.004; Fig. 3C). The animals in the ghrelin group did not achieve a significantly better test result compared to the animals in the control group (repeated-measures ANOVA, *p* = 0.08; Fig. 3C). Evaluation of the cylinder test yielded similar results. There was a significant reduction in motor deficits in rats treated with ghrelin compared to rats treated with the antagonist (repeated-measures ANOVA, *p* = 0.02; Fig. 3D) or vehicle (repeated-measures ANOVA, *p* = 0.04; Fig. 3D).

### Ghrelin modulates cognitive outcome after photothrombotic stroke

**Fig. 4:**
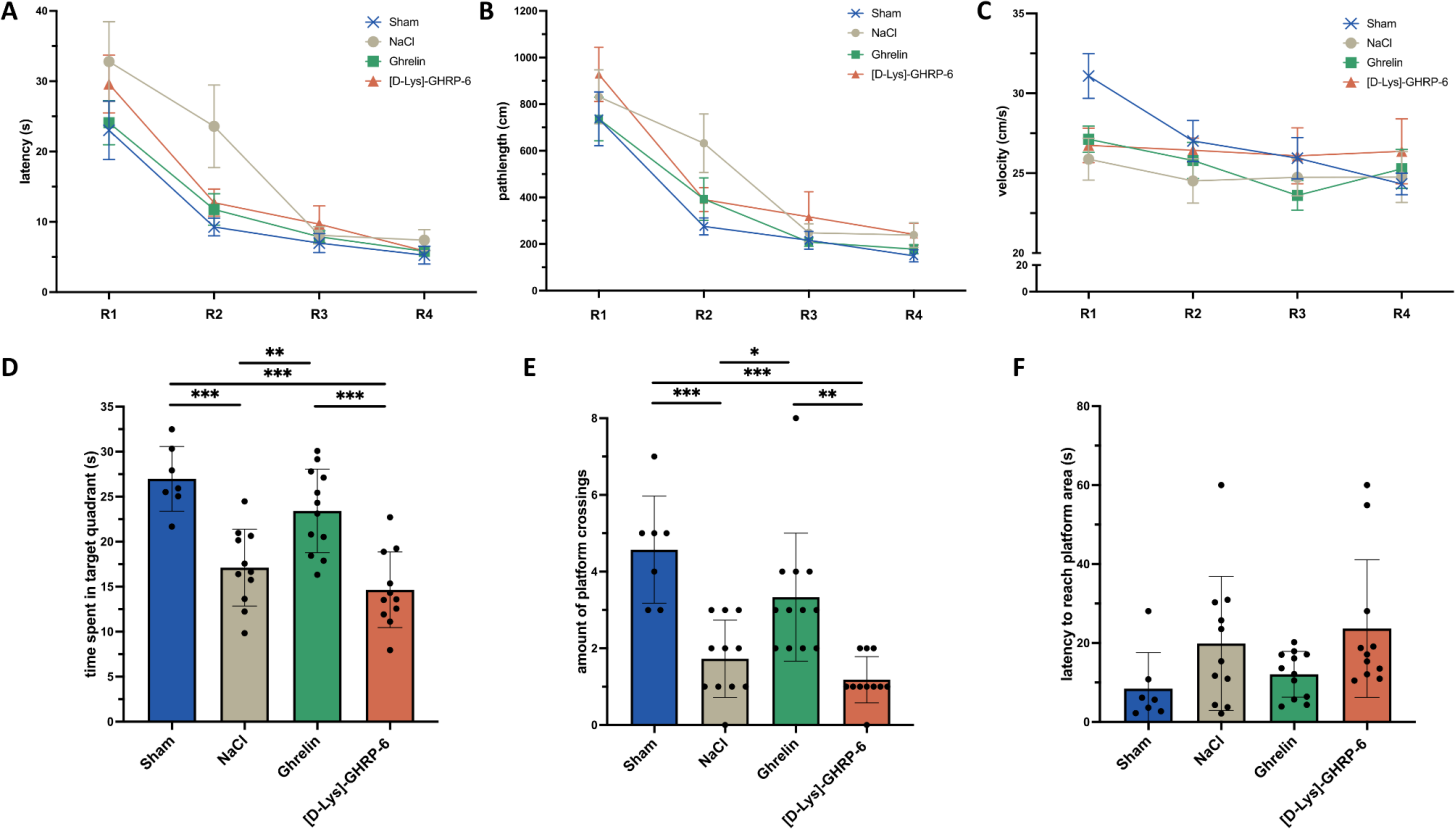
Spatial learning and memory formation after photothrombotic stroke. Assessment of memory acquisition: **(A)** Latency to reach the hidden platform, **(B)** path length, and **(C)** velocity. Assessment of memory retention: **(D)** time spent in the target quadrant, **(E)** number of platform crossings, and **(F)** latency to reach platform area. R: test run; One-way ANOVA followed by Fisher’s LSD; *P < 0.05, **P < 0.01, ***P < 0.001

In addition to sensorimotor recovery, cognitive impairment and recovery was next assessed using the Morris water maze test. During the acquisition trials, the distance traveled as well as the latency to reach the platform did not differ between the ghrelin, vehicle or antagonist treated rats and the sham-operated animals (repeated-measures ANOVA, *p* > 0.05 for all comparisons, Fig. 4A, B). Swimming speed did not differ between groups (repeated-measures ANOVA, *p* > 0.05 for all comparisons, Fig. 4C). The probe trial on day 19 after ischemia showed that ghrelin enhanced the retrieval of acquired memories. The time spent in the target quadrant was significantly longer in ghrelin-treated rats than in vehicle-treated animals (one-way ANOVA, *p* = 0.001; Fig. 4D) and ghrelin antagonist-treated animals (one-way ANOVA, *p* < 0.001; Fig. 4D). Ghrelin-treated rats crossed the platform area significantly more often compared to vehicle-treated animals (one-way ANOVA, *p* = 0.02; Fig. 4E) and ghrelin antagonist-treated animals (one-way ANOVA, *p* < 0.001; Fig. 4E). Latency to reach target showed no differences between groups (repeated-measures ANOVA; *p* > 0.05 for all comparisons, Fig. 4F). Again, velocity did not differ between groups (one-way ANOVA, *p* > 0.05 for all comparisons, data not shown), indicating that the results of the Water Maze test were not confounded by gross motor deficits.

### Ghrelin stimulates neurogenesis in the dentate gyrus, the subventricular zone and the peri-infarct region after photothrombotic stroke

**Fig. 5:**
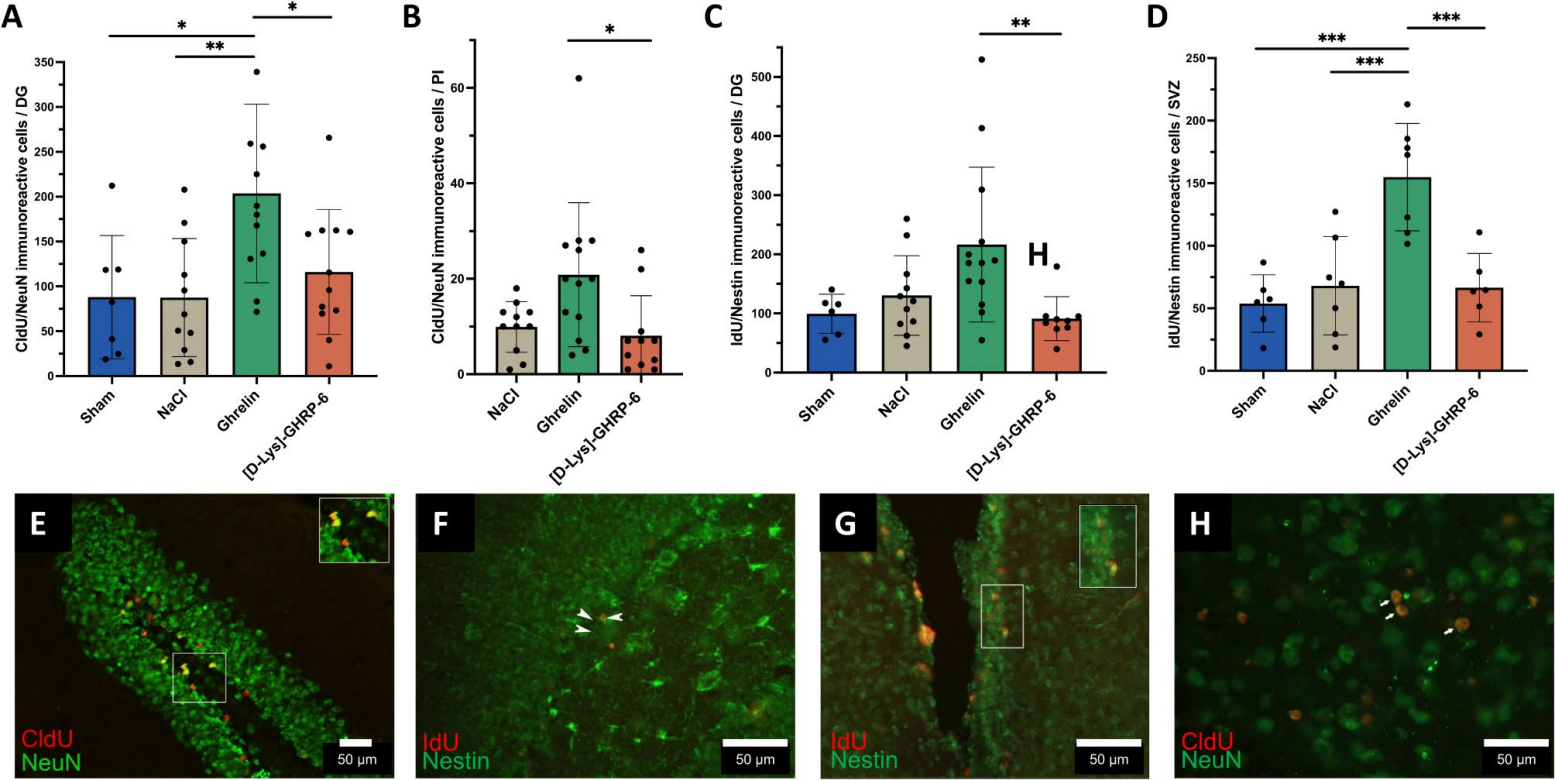
Analysis of neurogenesis after photothrombotic stroke. **(A)** Quantification of neurogenesis by detection of CldU/NeuN-expressing cells in the dentate gyrus (DG) and **(B)** peri-infarct area (PI). **(C)** Quantification of neurogenesis by detection of IdU/Nestin-expressing cells in the DG and **(D)** subventricular zone (SVZ). One-way ANOVA followed by Fisher’s LSD; *P < 0.05, **P < 0.01, ***P < 0.001. Representative photomicrographs of ghrelin-treated rats from the DG **(E**,** F)** and SVZ **(G)** demonstrating CldU/NeuN- **(E)** and IdU/Nestin-expressing cells **(F**,** G)**. Representative micrographs of ghrelin-treated rats from the peri-infarct area showing CldU/NeuN-expressing cells **(H)**

To investigate the mechanisms underlying the observed sensorimotor and cognitive recovery following ghrelin treatment in photothrombotic stroke, we evaluated neural progenitor cell proliferation in the peri-infarct region and established neurogenic niches, such as the SVZ of the lateral ventricle and the DG. To analyze the time course of new neuronal precursor cell generation, animals were treated with CldU and IdU to label cells of different chronological origins and were euthanized at 28 days after lesion induction, respectively. The combination staining of CldU and NeuN was used to detect cells that were newly generated and differentiated into neurons within 28 days after the stroke (Fig. 5E). The number of CldU^+^/NeuN^+^ immunoreactive cells was significantly higher in the DG of animals treated with ghrelin compared to those treated with vehicle (one-way ANOVA, *p* = 0.001; Fig. 5A), rats treated with the ghrelin receptor antagonist (one-way ANOVA, *p* = 0.005; Fig. 5A), and sham-operated animals (one-way ANOVA, *p* = 0.002; Fig. 5A). In the peri-infarct region, the number of newly generated cells was significantly increased in the ghrelin group compared to rats receiving the ghrelin receptor antagonist (Kruskal-Wallis test, *p* = 0.04; Fig. 5B, H). Next, we stained for IdU together with the stem cell marker Nestin to detect cells that proliferated shortly before perfusion and have the potential to develop neurons and glial cells (Fig. 5F, G). Significantly increased numbers of IdU-labeled neuronal precursor cells expressing Nestin were detected in the dentate gyrus of rats treated with ghrelin compared to those treated with the ghrelin receptor antagonist (Kruskal-Wallis test, *p* = 0.007; Fig. 5C). In the SVZ, the absolute number of double-positive cells differed significantly between animals treated with ghrelin compared to vehicle-treated animals (one-way ANOVA, *p* = 0.001; Fig. 5D), rats treated with the ghrelin receptor antagonist (one-way ANOVA, *p* = 0.001; Fig. 5D) and sham-operated animals (one-way ANOVA, *p* = 0.001; Fig. 5D). Taken together, these results show that ghrelin treatment after photothrombotic stroke induces sustained neurogenesis and proliferation of neural precursor cells within the peri-infarct region and neurogenic niches.

### Ghrelin does not modulate angiogenesis after photothrombotic stroke

To investigate the effects of the different treatment groups on angiogenesis, we assessed the length of blood vessels at the ischemic border zone. The results showed that there was no significant difference in vessel length between the groups (one-way ANOVA, *p* = 0.35; Suppl. Figure 1), indicating that the neuroregenerative properties of ghrelin are not associated to angiogenesis.

## Discussion

In the present study, ghrelin treatment was shown to improve sensorimotor and cognitive functions after experimental stroke. The gradual functional recovery during long-term follow-up and the marked improvement four weeks after treatment suggest a true recovery-promoting effect of ghrelin after stroke. We found increased neurogenesis in the dentate gyrus during the early postischemic phase of 1 to 4 days and in the subventricular zone and dentate gyrus during the postischemic phase of 25 to 28 days to be a mechanism contributing to this more favorable neurological outcome. Furthermore, ghrelin treatment had a positive effect on the number of newly generated neurons in the peri-infarct area at 28 days post-ischemia. In contrast to the neuroregenerative properties, no neuroprotective effects were found in the targeted neuroprotection studies with ghrelin treatment. Furthermore, there were no effects of ghrelin treatment on postischemic angiogenesis in the peri-infarction area.

Here, for the first time, we systematically examined whether exogenous ghrelin affects long-term functional and structural recovery after ischemic stroke in combination with early outcome assessments. Previous studies showing improved outcomes after ghrelin treatment mainly focused on the first three days after stroke induction. These studies suggested that acylated and desacylated ghrelin had neuroprotective properties in experimental models of stroke when administered before induction of cerebral ischemia or shortly after induction of reperfusion [23, 24, 26, 29, 30]. In contrast, we and others [25, 26], have demonstrated a limited capacity of ghrelin’s neuroprotective actions. In our study, the observed improvement in functional recovery in ghrelin-treated animals was not due to a reduction in infarct volume, suggesting a true recovery effect of ghrelin rather than neuroprotective actions. With regard to ghrelin-induced neuroprotection, the time of administration may play an important role. In many studies demonstrating neuroprotective effects in vivo, ghrelin was administered before stroke induction or during ischemia and before reperfusion [23, 24, 26]. Obviously, early treatment with ghrelin is beneficial due to its neuroprotective effect. In line with the current STAIR recommendations, further ambitious clinical studies could help to confirm the evidence and prepare for translation. The aim should be to investigate the administration of ghrelin shortly after the onset of stroke in combination with reperfusion in order to determine the optimal conditions for its acute application in ischemia-reperfusion models [31].

In the present study, ghrelin treatment resulted in a significant improvement in spatial information retrieval eight days after the last training session in the water maze test. Consistent with our previous study, experimental photothrombotic stroke did not affect spatial memory acquisition, whereas long-term retention of acquired memory was significantly impaired [32]. Ghrelin treatment specifically restored brain damage. However, antagonizing endogenous ghrelin in vehicle-treated animals with the ghrelin receptor antagonist [D-Lys(3)]GHRP-6 did not result in an exacerbation of the cognitive deficit, suggesting a receptor-independent mechanism of action. This is in line with previous studies showing an improvement in short- and long-term memory after ghrelin administration [9, 21, 33]. Remarkably, memory deficits in ghrelin-deficient mice were reversed by ghrelin replacement [34].

Regeneration after stroke is a complex and multifaceted process. It involves synaptic plasticity, which allows the brain to adapt and reorganize despite neuronal loss. Stroke-induced plasticity can lead to the reorganization of functional networks, particularly in the peri-infarcted region, thereby promoting the recovery of motor and cognitive function [35]. In addition, axonal sprouting and the formation of new synaptic connections help to restore communication between neurons [36]. Inflammation is a critical aspect of stroke repair. Microglia and infiltrating macrophages are activated early after ischemia and release cytokines and chemokines that may exacerbate neuronal damage but also promote tissue repair [37]. Regulating the balance between pro- and anti-inflammatory responses can influence the overall recovery process. Ghrelin has been shown to possess anti-inflammatory properties, including by regulating microglial activity during neuroinflammation [38], which may complement its role in promoting neurogenesis.

Endogenous neurogenesis is thought to play a significant role in mediating functional and especially cognitive recovery after ischemic stroke [39, 40]. Ischemic stroke stimulates the proliferation of neural precursor cells in the SVZ and DG, as well as the migration of newborn immature neurons from the SVZ to the lesioned areas [41–44]. Endogenous neurogenesis is likely to be a crucial mechanism for the remarkable capacity of the brain for self-repair, but it is insufficient to reverse brain damage after stroke. Several therapeutically active compounds have been shown to promote neurogenesis and alleviate motor and cognitive deficits after experimental stroke [20]. Since most newborn cells normally die before maturation, it is important to promote the survival of these cells in order to effectively support endogenous neurogenesis as a regenerative mechanism after stroke. In our study, we used two different thymidine analogues, CldU and IdU, to label two different subpopulations of newborn cells of different ages and developmental stages. These analogues can be clearly distinguished from each other using appropriate antibodies and label two populations of different ages in the same animal. In our study, ghrelin treatment resulted in increased cell survival in the peri-infarct region and dentate gyrus that arose in the first four days after stroke. Ghrelin treatment also resulted in a sustained increase in neural progenitor cells up to 28 days after stroke.

Despite correlative studies, there is strong evidence for a causal link between neurogenesis and improved recovery after stroke. The removal of neural precursor cells expressing doublecortin prior to permanent cerebral ischemia in a transgenic mouse model expressing doublecortin-thymidine kinase led to excessive postischemic sensorimotor deficits [45]. Furthermore, it has been shown that the conditional ablation of neuroprogenitor cells hinders the restoration of cognitive function after a stroke and reduces synaptic connectivity [46]. Therefore, the attenuation of sensorimotor and cognitive declines by ghrelin in association with enhanced neurogenesis strongly suggests the neuroregenerative properties of this therapy. However, the exact molecular mechanisms of ghrelin treatment for ischemic stroke remain to be elucidated. In the present study, there were no significant differences in neurogenesis and neural progenitor cell proliferation in control animals compared to those receiving the ghrelin receptor antagonist. Thus, the beneficial effects of ghrelin do not appear to be solely due to its action on GHS-R1a, as the blocking therapy was not associated with a significant reduction in neurogenesis. These results are consistent with those of Johansson and colleagues, who demonstrated proliferative effects on cultured hippocampal progenitor cells that did not express GHS-R1a [14].

It is important to note that our study is limited by the inclusion of only healthy male animals at a relatively young age. It is therefore essential to extend this study to female animals, as well as older animals and animals with comorbidities (e.g. hypertension). This will allow a solid evaluation of the therapeutic potential proposed here and its potential applicability in clinical settings. A potential limitation of our data interpretation is the use of two different animal models of stroke, each used for a different outcome parameter, acute vs. chronic phase. Therefore, we cannot determine whether the observed effects are consistent across both models. From a translational perspective, the use of different animal models of stroke is a valuable approach that offers unique insights. The use of the MCAO and photothrombotic models, each with its own strengths and weaknesses, made it possible to minimize the systematic errors associated with each model and gain a more comprehensive understanding of the effectiveness of an intervention.

The results of this study contribute to the expanding body of research on stroke recovery, particularly with regard to the role of ghrelin in this process. However, it is important to situate these findings in the broader context of stroke recovery, which involves multiple interacting processes. An understanding of how ghrelin may influence these various mechanisms may further elucidate its potential as a multifaceted therapeutic approach for stroke patients.

## Conclusions

In conclusion, the present data demonstrate that ghrelin is a promising candidate for a therapeutic agent, particularly in the subacute and chronic phase after ischemic stroke. It alleviates sensorimotor and cognitive deficits and promotes endogenous repair mechanisms by enhancing long-term survival and sustainable proliferation of newborn neurons in the peri-infarcted region as well as in the hippocampus. In contrast to previous data, ghrelin has no significant neuroprotective effects in acute ischemic stroke.

## Electronic supplementary material

Below is the link to the electronic supplementary material.

Supplementary Material 1

## Data Availability

The data that support the findings of this study are available on request from the corresponding author.

## Abbreviations

ANOVA: Analysis of variance
CldU: Chlorodeoxyuridine
DG: Dentate gyrus
IdU: Iododeoxyuridine
LSD: Least significant difference
MCAO: Middle cerebral artery occlusion
NeuN: Neuronal nuclear protein
NS/PCs: Neural stem/progenitor cells
SVZ: Subventricular zone

## References

[1] Benjamin, E. J., Blaha, M. J., Chiuve, S. E., et al. (2017). Heart Disease and Stroke Statistics-2017 update: A Report from the American Heart Association. *Circulation*, *135*, e146–603. 10.1161/CIR.0000000000000485 28122885 10.1161/CIR.0000000000000485 PMC5408160

[2] Levine, D. A., Galecki, A. T., Langa, K. M., et al. (2015). Trajectory of cognitive decline after Incident Stroke. *Journal of the American Medical Association*, *314*, 41–51. 10.1001/jama.2015.6968 26151265 10.1001/jama.2015.6968 PMC4655087

[3] Pendlebury, S. T., & Rothwell, P. M. (2009). Prevalence, incidence, and factors associated with pre-stroke and post-stroke dementia: A systematic review and meta-analysis. *Lancet Neurology*, *8*, 1006–1018. 10.1016/S1474-4422(09)70236-4 19782001 10.1016/S1474-4422(09)70236-4

[4] Chen, D., Wei, L., Liu, Z-R., et al. (2018). Pyruvate kinase M2 increases Angiogenesis, Neurogenesis, and functional recovery mediated by Upregulation of STAT3 and focal adhesion kinase activities after ischemic stroke in adult mice. *Neurotherapeutics*, *15*, 770–784. 10.1007/s13311-018-0635-2 29869055 10.1007/s13311-018-0635-2 PMC6095793

[5] Belayev, L., Hong, S-H., Menghani, H., et al. (2018). Docosanoids Promote Neurogenesis and Angiogenesis, Blood-Brain Barrier Integrity, Penumbra Protection, and Neurobehavioral Recovery after experimental ischemic stroke. *Molecular Neurobiology*, *55*, 7090–7106. 10.1007/s12035-018-1136-3 29858774 10.1007/s12035-018-1136-3 PMC6054805

[6] Madelaine, R., Sloan, S. A., Huber, N., et al. (2017). MicroRNA-9 couples brain neurogenesis and angiogenesis. *Cell Rep*, *20*, 1533–1542. 10.1016/j.celrep.2017.07.051 28813666 10.1016/j.celrep.2017.07.051 PMC5665055

[7] Abizaid, A., Liu, Z-W., Andrews, Z. B., et al. (2006). Ghrelin modulates the activity and synaptic input organization of midbrain dopamine neurons while promoting appetite. *J Clin Invest*, *116*, 3229–3239. 10.1172/JCI29867 17060947 10.1172/JCI29867 PMC1618869

[8] Naleid, A. M., Grace, M. K., Cummings, D. E., et al. (2005). Ghrelin induces feeding in the mesolimbic reward pathway between the ventral tegmental area and the nucleus accumbens. *Peptides*, *26*, 2274–2279. 10.1016/j.peptides.2005.04.025 16137788 10.1016/j.peptides.2005.04.025

[9] Diano, S., Farr, S. A., Benoit, S. C., et al. (2006). Ghrelin controls hippocampal spine synapse density and memory performance. *Nature Neuroscience*, *9*, 381–388. 10.1038/nn1656 16491079 10.1038/nn1656

[10] Gahete, M. D., Córdoba-Chacón, J., Kineman, R. D., et al. (2011). Role of ghrelin system in neuroprotection and cognitive functions: Implications in Alzheimer’s disease. *Peptides*, *32*, 2225–2228. 10.1016/j.peptides.2011.09.019 21983104 10.1016/j.peptides.2011.09.019 PMC3228413

[11] Tian, J., Wang, T., & Du, H. (2023). Ghrelin system in Alzheimer’s disease. *Current Opinion in Neurobiology*, *78*, 102655. 10.1016/j.conb.2022.102655 36527939 10.1016/j.conb.2022.102655 PMC10395051

[12] Andrews, Z. B. (2011). The extra-hypothalamic actions of ghrelin on neuronal function. *Trends in Neurosciences*, *34*, 31–40. 10.1016/j.tins.2010.10.001 21035199 10.1016/j.tins.2010.10.001

[13] Spencer, S. J., Miller, A. A., & Andrews, Z. B. (2013). The role of Ghrelin in Neuroprotection after Ischemic Brain Injury. *Brain Sci*, *3*, 344–359. 10.3390/brainsci3010344 24961317 10.3390/brainsci3010344 PMC4061836

[14] Johansson, I., Destefanis, S., Åberg, N. D., et al. (2008). Proliferative and protective effects of growth hormone secretagogues on adult rat hippocampal progenitor cells. *Endocrinology*, *149*, 2191–2199. 10.1210/en.2007-0733 18218693 10.1210/en.2007-0733

[15] Moon, M., Kim, S., Hwang, L., et al. (2009). Ghrelin regulates hippocampal neurogenesis in adult mice. *Endocrine Journal*, *56*, 525–531. 10.1507/endocrj.k09e-089 19506321 10.1507/endocrj.k09e-089

[16] Dirnagl, U., Iadecola, C., & Moskowitz, M. A. (1999). Pathobiology of ischaemic stroke: An integrated view. *Trends in Neurosciences*, *22*, 391–397. 10.1016/s0166-2236(99)01401-0 10441299 10.1016/s0166-2236(99)01401-0

[17] Ohab, J. J., & Carmichael, S. T. (2008). Poststroke neurogenesis: Emerging principles of migration and localization of immature neurons. *Neurosci Rev J Bringing Neurobiol Neurol Psychiatry*, *14*, 369–380. 10.1177/1073858407309545 10.1177/1073858407309545 18024854

[18] Wahl, A. S., Omlor, W., Rubio, J. C., et al. (2014). Neuronal repair. Asynchronous therapy restores motor control by rewiring of the rat corticospinal tract after stroke. *Science*, *344*, 1250–1255. 10.1126/science.1253050 24926013 10.1126/science.1253050

[19] Liang, H., Zhao, H., Gleichman, A., et al. (2019). Region-specific and activity-dependent regulation of SVZ neurogenesis and recovery after stroke. *Proceedings of National Academy of Sciences*, *116*, 13621–13630. 10.1073/pnas.1811825116 10.1073/pnas.1811825116 PMC6612913 31196958

[20] Rahman, A. A., Amruta, N., Pinteaux, E., et al. (2021). Neurogenesis after stroke: A therapeutic perspective. *Transl Stroke Res*, *12*, 1–14. 10.1007/s12975-020-00841-w 32862401 10.1007/s12975-020-00841-w PMC7803692

[21] Carlini, V. P., Ghersi, M., Schiöth, H. B., et al. (2010). Ghrelin and memory: Differential effects on acquisition and retrieval. *Peptides*, *31*, 1190–1193. 10.1016/j.peptides.2010.02.021 20214944 10.1016/j.peptides.2010.02.021

[22] Kent, B. A., Beynon, A. L., Hornsby, A. K. E., et al. (2015). The orexigenic hormone acyl-ghrelin increases adult hippocampal neurogenesis and enhances pattern separation. *Psychoneuroendocrinology*, *51*, 431–439. 10.1016/j.psyneuen.2014.10.015 25462915 10.1016/j.psyneuen.2014.10.015 PMC4275579

[23] Miao, Y., Xia, Q., Hou, Z., et al. (2007). Ghrelin protects cortical neuron against focal ischemia/reperfusion in rats. *Biochemical and Biophysical Research Communications*, *359*, 795–800. 10.1016/j.bbrc.2007.05.192 17560544 10.1016/j.bbrc.2007.05.192

[24] Hwang, S., Moon, M., Kim, S., et al. (2009). Neuroprotective effect of ghrelin is associated with decreased expression of prostate apoptosis response-4. *Endocrine Journal*, *56*, 609–617. 10.1507/endocrj.k09e-072 19352052 10.1507/endocrj.k09e-072

[25] Ku, J. M., Taher, M., Chin, K. Y., et al. (2016). Protective actions of Des-acylated ghrelin on brain injury and blood-brain barrier disruption after stroke in mice. *Clin Sci Lond Engl 1979*, *130*, 1545–1558. 10.1042/CS20160077 10.1042/CS20160077 27303049

[26] Chung, H., Li, E., Kim, Y., et al. (2013). Multiple signaling pathways mediate ghrelin-induced proliferation of hippocampal neural stem cells. *Journal of Endocrinology*, *218*, 49–59. 10.1530/JOE-13-0045 23608221 10.1530/JOE-13-0045

[27] Carmichael, S. T. (2005). Rodent models of focal stroke: Size, mechanism, and purpose. *NeuroRX*, *2*, 396–409. 10.1602/neurorx.2.3.396 16389304 10.1602/neurorx.2.3.396 PMC1144484

[28] Urbaniak, G. C., & Plous, S. (2013). *Research randomizer (version 4.0) [Computer software]*. Retrieved June 22, 2013, from http://www.randomizer.org/

[29] Chung, H., Kim, E., Lee, D. H., et al. (2007). Ghrelin inhibits apoptosis in hypothalamic neuronal cells during oxygen-glucose deprivation. *Endocrinology*, *148*, 148–159. 10.1210/en.2006-0991 17053024 10.1210/en.2006-0991

[30] Liu, Y., Wang, P. S., Xie, D., et al. (2006). Ghrelin reduces injury of hippocampal neurons in a rat model of cerebral ischemia/reperfusion. *Chinese Journal of Physiology*, *49*, 244–250. 17294832

[31] Wechsler, L. R., Adeoye, O., Alemseged, F., et al. (2023). Most promising approaches to improve stroke outcomes: The Stroke Treatment Academic Industry Roundtable XII workshop. *Stroke*, *54*, 3202–3213. 10.1161/STROKEAHA.123.044279 37886850 10.1161/STROKEAHA.123.044279

[32] Diederich, K., Schmidt, A., Strecker, J-K., et al. (2014). Cortical photothrombotic infarcts impair the recall of previously acquired memories but spare the formation of new ones. *Stroke*, *45*, 614–618. 10.1161/STROKEAHA.113.001907 24347420 10.1161/STROKEAHA.113.001907

[33] Carlini, V. P., Monzón, M. E., Varas, M. M., et al. (2002). Ghrelin increases anxiety-like behavior and memory retention in rats. *Biochemical and Biophysical Research Communications*, *299*, 739–743. 10.1016/s0006-291x(02)02740-7 12470640 10.1016/s0006-291x(02)02740-7

[34] Li, E., Chung, H., Kim, Y., et al. (2013). Ghrelin directly stimulates adult hippocampal neurogenesis: Implications for learning and memory. *Endocrine Journal*, *60*, 781–789. 10.1507/endocrj.ej13-0008 23411585 10.1507/endocrj.ej13-0008

[35] Joy, M. T., & Carmichael, S. T. (2021). Encouraging an excitable brain state: Mechanisms of brain repair in stroke. *Nature Reviews Neuroscience*, *22*, 38–53. 10.1038/s41583-020-00396-7 33184469 10.1038/s41583-020-00396-7 PMC10625167

[36] Carmichael, S. T., Kathirvelu, B., Schweppe, C. A., et al. (2017). Molecular, cellular and functional events in axonal sprouting after stroke. *Experimental Neurology*, *287*, 384–394. 10.1016/j.expneurol.2016.02.007 26874223 10.1016/j.expneurol.2016.02.007 PMC4980303

[37] Lambertsen, K. L., Finsen, B., & Clausen, B. H. (2019). Post-stroke inflammation-target or tool for therapy? *Acta Neuropathol (Berl)*, *137*, 693–714. 10.1007/s00401-018-1930-z 30483945 10.1007/s00401-018-1930-z PMC6482288

[38] Ma, Y., Zhang, H., Guo, W., et al. (2022). Potential role of ghrelin in the regulation of inflammation. *The Faseb Journal*, *36*, e22508. 10.1096/fj.202200634R 35983825 10.1096/fj.202200634R

[39] Arvidsson, A., Collin, T., Kirik, D., et al. (2002). Neuronal replacement from endogenous precursors in the adult brain after stroke. *Nature Medicine*, *8*, 963–970. 10.1038/nm747 12161747 10.1038/nm747

[40] Lagace, D. C. (2012). Does the endogenous neurogenic response alter behavioral recovery following stroke? *Behavioural Brain Research*, *227*, 426–432. 10.1016/j.bbr.2011.08.045 21907736 10.1016/j.bbr.2011.08.045

[41] Zhang, R. L., Zhang, Z. G., Zhang, L., et al. (2001). Proliferation and differentiation of progenitor cells in the cortex and the subventricular zone in the adult rat after focal cerebral ischemia. *Neuroscience*, *105*, 33–41. 10.1016/s0306-4522(01)00117-8 11483298 10.1016/s0306-4522(01)00117-8

[42] Liu, J., Solway, K., Messing, R. O., et al. (1998). Increased neurogenesis in the dentate gyrus after transient global ischemia in gerbils. *J Neurosci off J Soc Neurosci*, *18*, 7768–7778. 10.1523/JNEUROSCI.18-19-07768.1998 PMC6793017 9742147

[43] Lindvall, O., & Kokaia, Z. (2015). Neurogenesis following stroke affecting the adult brain. *Cold Spring Harbor Perspectives in Biology*, *7*, a019034. 10.1101/cshperspect.a019034 26525150 10.1101/cshperspect.a019034 PMC4632663

[44] Yamashita, T., Ninomiya, M., Hernández Acosta, P., et al. (2006). Subventricular zone-derived neuroblasts migrate and differentiate into mature neurons in the post-stroke adult striatum. *J Neurosci off J Soc Neurosci*, *26*, 6627–6636. 10.1523/JNEUROSCI.0149-06.2006 10.1523/JNEUROSCI.0149-06.2006 PMC6674034 16775151

[45] Jin, K., Wang, X., Xie, L., et al. (2010). Transgenic ablation of doublecortin-expressing cells suppresses adult neurogenesis and worsens stroke outcome in mice. *Proc Natl Acad Sci U S A*, *107*, 7993–7998. 10.1073/pnas.1000154107 20385829 10.1073/pnas.1000154107 PMC2867852

[46] Sun, C., Sun, H., Wu, S., et al. (2013). Conditional ablation of Neuroprogenitor cells in adult mice impedes recovery of Poststroke cognitive function and reduces synaptic connectivity in the Perforant Pathway. *Journal of Neuroscience*, *33*, 17314–17325. 10.1523/JNEUROSCI.2129-13.2013 24174664 10.1523/JNEUROSCI.2129-13.2013 PMC3812503

