# Supplementary material for: Ghrelin promotes neurologic recovery and neurogenesis in the chronic phase after experimental stroke
Source: Neurol Res Pract. 2025 Mar 3;7(1):14. doi: 10.1186/s42466-025-00371-6 (PMC11921976; doi:10.1186/s42466-025-00371-6)

Supplementary Material

# Supplementary Methods

**Sample size calculation**

Sample size was determined *a priori* by performing a power analysis (Power = 0.8, alpha error probability=0.05, G*Power 3.1.9.7). The sample size for Experiment 1 (postischemic neuroprotection) was determined by a power analysis with a targeted effect size of 1.2. The primary endpoint was infarct volume. Given that two treatment groups were to be compared, a t-test was designated as the statistical test. Sample size for experiment 2 (postischemic regeneration) was determined by a power analysis with a targeted effect size of 0.2. The primary endpoint was sensorimotor performance as measured by the adhesive removal test. Given that four treatment groups were to be compared, an ANOVA with repeated measures was designated as the statistical test.

**Middle cerebral artery occlusion**

Middle cerebral artery occlusion (MCAO) was initiated in rats under anesthesia with an intraperitoneal injection of ketamine hydrochloride (100 mg/kg body weight; Ketanest) and xylazine hydrochloride (8 mg/kg body weight), maintaining a constant body temperature of 37˚C ± 0.5˚C throughout the procedure. All animals received meloxicam (1 mg/kg bw s.c.) 30 min prior to surgery. After a midline neck incision, the left common carotid artery and carotid bifurcation were exposed, and the proximal left common and external carotid arteries were ligated. Retrograde perfusion of the left common carotid artery was temporarily interrupted by a microvascular clip (FE691; Aesculap), the common carotid artery was incised, and the occluder (made from a 2.5 cm long 4.0 nylon suture (Ethilon, Tilburg, the Netherlands) rounded to a diameter of 0.2-0.3 mm using a soldering iron) was inserted into the middle cerebral artery. After 45 minutes of MCAO verified by laser Doppler (Periflux 5001; Perimed), the monofilament was withdrawn to allow reperfusion of the middle cerebral artery. After the procedure, the wound was sutured and treated with xylocain gel (2%). For postoperative analgesia, animals received meloxicam (1 mg/kg s.c.) for 3 days with the first dose administered during surgery under anesthesia. The same procedure was used in sham animals, except that the nylon filament was retracted immediately after insertion.

**Photothrombotic Stroke**

To induce photothrombotic cortical stroke, rats were anesthetized with an intraperitoneal injection of ketamine hydrochloride (100 mg/kg body weight; Ketanest) and xylazine hydrochloride (8 mg/kg body weight). Animals received meloxicam (1 mg/kg bw s.c.) 30 min prior to surgery. The body temperature of 36.5 +/- 0.5 °C was maintained throughout the procedure and for 2 hours afterwards. The left femoral vein was cannulated with a PE-50 tube for Bengal Rose infusion. Rectal temperature was maintained at 37°C by a thermostatically controlled heating pad (Föhr Medical Instruments, Germany). Photothrombotic ischemia was induced in the right frontal cortex. For illumination, an 8-mm-diameter laser spot (Cobolt Jive ^TM^ 75 Laser, 561 nm, Cobolt AB Schweden) was placed stereotaxically onto the skull, 0.5 mm anterior to the bregma and 3.5 mm lateral to the midline. The skull was illuminated for 20 minutes. During the first 2 minutes of illumination, Bengal Rose dye (0.133 mL/kg body weight, 10 mg/mL saline) was injected intravenously. After the procedure, the wound was sutured and treated with xylocain gel (2%). For postoperative analgesia, animals received meloxicam (1 mg/kg s.c.) for 3 days with the first dose administered during surgery under anesthesia. Sham-operated animals were subjected to the same procedure, including Bengal Rose injection, but without illumination of the skull.

**Experimental Design**

In experiment 1, we examined the effect of ghrelin on early postischemic neuroprotection **(Figure 1A)**. MCAO was induced in rats for 45 min. Rats were injected subcutaneously with either vehicle control substance (1 ml NaCl 0.9%, n=12) or acylated ghrelin (no. 031-31; Phoenix Pharmaceuticals, Inc. Belmont, CA, USA, 80 μg/kg body weight, n=12) 1 hour after induction of MCAO. Rotarod test was performed to compare sensorimotor deficits. Twenty-four hours after MCAO, rats were perfused, and brains were harvested. Infarct volume was calculated to assess structural damage. The attrition rate as a consequence of complications associated with the surgical procedure as well as the number of subjects excluded from individual analysis and the reasons for exclusions, are provided in Suppl. Figure 2.

In experiment 2, we examined the effect of ghrelin on postischemic neuroregeneration **(Figure 1B)**. Photothrombosis was induced on day 0, and animals were randomly assigned to one of the following treatment groups: (1) sham-operated rats (n=7), (2) vehicle treatment (1 ml NaCl 0.9%, n=11), (3) acylated ghrelin (no. 031-31, Phoenix Pharmaceuticals, Inc. Belmont, USA, 60 μg/kg body weight, n=14), or (4) ghrelin receptor antagonist [D-Lys]-GHRP-6 (031-22, Phoenix Pharmaceuticals, Inc. Belmont, USA, 2 mg/kg, n=12). Depending on which intervention group they belonged to, animals received a daily dose of vehicle, ghrelin, or [D-Lys]-GHRP-6 subcutaneously via a neck-mounted micro-osmotic pump for 28 days. The micro-osmotic pumps (Model 1004, Alzet Osmotic Pumps, Cupertino, USA) were implanted immediately after photothrombosis. The dose of ghrelin (60 μg/kg per day) or [D-Lys]-GHRP-6 (2mg/kg per day) was dissolved in 0.9% NaCl and filled into the pumps (reservoir volume 100 μl). The therapeutic substances were continuously administered into the subcutaneous tissue with an hourly injection volume of 0.11 μl, starting 24 hours after implantation and throughout the experimental period of 28 days. A lower dosage for Ghrelin was selected for continuous administration over 28 days via the osmotic pump to facilitate uniform distribution, maintain a consistent drug concentration, and circumvent potential adverse effects associated with peak concentrations.

On days 1-4 after induction of ischemia or sham treatment, animals received intraperitoneally the thymidine analogue chlorodeoxyuridine (CldU, 50µg/kg), and on days 25-28 after ischemia, the thymidine analogue iododeoxyuridine (IdU, 50µg/kg) **(Figure 1C**), which can be detected separately by immunohistochemistry. In addition, a battery of somatosensory tests was performed on day −1 (baseline) and days 1, 3, 7, 14, 21 and 28. This test battery consisted of the cylinder test and the adhesive tape removal test. We also performed the Morris water maze test during the second week after photothrombosis. After 28 days, the animals were perfused, and the brains were harvested for histological analyses. The attrition rate as a consequence of complications associated with the surgical procedure as well as the number of subjects excluded from individual analysis and the reasons for exclusions, are provided in Suppl. Figure 2.

**Behavioral assessment**

*Neuroscore*

To analyze the overall functional outcome, a modification of the Menzies neuroscore was used, ranging from 0 (no deficit) to 5 (death). Depending on the condition of the animal, a neuroscore of 0 (healthy animal), 1 (flexion of the contralateral forepaw), 2 (reduced strength of the forepaws), 3 (turning in the air in the ipsilateral direction after lifting the tail), 4 (animal spontaneously turns in a circle) or 5 (animal died) was recorded.

*Assessment of sensorimotor performance*

A baseline test was performed in all animals 1 day before ischemia induction.

The rotarod tests were performed by placing rats on an accelerating rotarod cylinder, and the time the animals remained on the rotarod was measured. Speed was increased from 4 to 40 rpm within 300 seconds. The trial ended if the animal fell off the rungs or gripped the device and spun around for 2 consecutive revolutions without attempting to walk on the rungs. An arbitrary time limit of 300 seconds was set for rats on the Rotarod cylinder in training and testing procedures. Prior to baseline testing, the animals were trained daily for 5 days on the procedure.

For the adhesive removal test, 2 pieces of adhesive-backed paper dots (113 mm diameter) were used as bilateral tactile stimuli occupying the palmar surface of each forepaw. The time to remove each paper dot from the forelimbs was documented in 3 trials per day for each forepaw and an average value was determined for each side. An asymmetry score was calculated as follows: (time to remove ipsilateral dot− time to remove contralateral dot)/(time to remove ipsilateral dot+ time to remove contralateral dot). Through daily training that began three days before ischemia induction, the animals were trained to remove the adhesive dots in less than 10 seconds.

For the cylinder test, the rats were placed in a transparent cylinder (16-cm diameter, 21-cm height) and videotaped. Spontaneous wall and ground touches of both forelimbs were counted, and an asymmetry score was calculated as described above.

The order of testing as well as the time of day of testing per animal was kept constant to minimize diurnal influences on testing. All tests were performed during the animals' light phase.

*Assessment of cognitive performance*

Within the Morris water maze test, animals learned to use spatial cues to find a hidden escape platform located at a fixed position below the water surface. On each day, 4 test runs were performed, with an interval of 60 seconds between each run. Animals were released into the pool from randomly varying positions for a maximum trial duration of 90 s. If the platform was not located, the animal was gently guided to the platform and allowed to re-orient to the spatial cues for 10 s before being removed from the pool. During the acquisition trials, the latency to reach the platform and the swimming speed were recorded using Ethovision XT tracking software (Noldus information technology, Wageningen, Netherlands). In the probe trial, which was performed without an escape platform, the time the animals navigated through the former platform quadrant and the amount of platform crossings were recorded. One day before the start of the acquisition phase, the animals were habituated to the experimental setup by placing them in the pool with a visible platform. Four test runs were performed.

**Immunohistochemistry following photothrombosis (experiment 2)**

Every 6th section (240~~-~~μm intervals) was selected from each animal and processed for immunohistochemistry. Mounted coronal brain cryosections (40μm) were rinsed in 3% H_2_O_2_/Methanol for 10 minutes to block endogenous peroxidases. Following extensive washes in PBS, sections were blocked with a solution containing PBS, 0.1% Triton-X100 (Carl Roth) and 3% Blocking Reagent (Roche Diagnostics) for 30 min to prevent nonspecific protein binding. For detection of IdU- and CldU-positive cells, sections were incubated in 50% Formamid/2xSSC followed by washes in 2x SSC. Then, the sections were placed in 0.65N HCl at 60°C for 20 min, followed by 0.1M borate buffer for 10 min. After that, the sections were blocked with a solution containing TBS, 0.1% Triton-X100 and 3% Blocking Reagent. Primary antibodies were applied overnight at 4°C. We used the following primary antibodies for murine sections: anti-NeuN (1:200; Millipore, Darmstadt, Germany), mouse anti-neuronal nuclei and rat anti-CD31 (1:250; Abcam), anti-CldU (1:500; Abcam, Cambridge, UK) anti-IdU (1:200; BD BioSciences, Cambridge, UK), and Nestin (1:500; Abcam, Cambridge, UK). Detection of anti-NeuN antibodies was done with a goat anti-mouse fluorescent dye (AlexaFluor488, 1:100, 45min.; Molecular Probes, Leiden, Netherlands). Bromodeoxyuridine antibodies were detected using a biotin conjugated goat-anti-rat antibody (1:500; 45min; Jackson Labs, West Grove, PA, USA); CD31-antibodies detection was done using a biotin conjugated goat-anti-rat antibody (1:100; 45min; Jackson Labs). For signal amplification of IdU-, CD31- and CldU-signal, sections were incubated with horse radish peroxidase/streptavidin (1:100, 45min; DAKO, Glostrup, Denmark) and biotinyl tyramide (1:100). IdU-, CD31- and CldU-positive cells were visualized by a streptavidin/fluorescent dye (AlexaFluor594, Molecular Probes). Nuclei counterstain was done with a tissue preserving medium containing 4’,6-diamidino-2-phenylindole (DAPI, Vector, Burlingame, CA, USA). Immunoflurescence was computed and visualized with a Nikon Eclipse 80i fluorescence microscope (Nikon, Düsseldorf, Germany) equipped with proper filter sets for AlexaFluor594, AlexaFluor488 and DAPI. Five stained sections per animal were examined for evaluation.

**Infarct volume assessment**

Calculation of infarct volume following MCAO or photothrombosis was done by collecting coronal brain cryosections (10 μm) every 240 μm starting at the rostral border of the infarct. The rostral border of the infarct was delineated on the unstained sections by the lightening of the tissue in comparison to the healthy brain parenchyma. Toluidine staining was performed to validate the infarct borders. For this purpose, the slices were stained with 0.5% toluidine blue (Sigma, St. Louis, MO) and then each was dried in graded ethanol for 1 minute (50%, 80%, 96%, 100%). Six tissue sections of a brain were used to assess the infarct volumes. Digitized images of infarct area and the areas of ipsilateral hemisphere and contralateral hemisphere were measured by a blinded investigator on each section using ImageJ software 1.48v. The final infarct volume was calculated by multiplying infarct area size by the distance to the next section.

**Assessment of neuroregenerative mechanisms**

*Analysis of Neurogenesis*

CldU-positive cells were assessed in the dentate gyrus (DG) of the hippocampus, and peri-infarct area on six serial sections spaced 240 μm apart. In the DG, CldU-positive cells were assessed manually in the ipsi- and contralesional hemisphere. Fifty randomly selected cells per counting site were analyzed for co-expression of CldU and NeuN for neuronal phenotype. CldU-positive cells in the peri-infarct area were analyzed in three frames (300 x 300 μm) adjacent to the peri-infarct area on the section depicting the maximal infarct expansion and in one section each 240 μm anterior and posterior. Each CldU-positive cell was also checked for NeuN co-expression.

IdU-positive cells were assessed in the DG, the subventricular zone (SVZ), and peri-infarct area. IdU-positive cells were analyzed analogously to CldU-positive cells. In the DG, IdU-positive cells were counted manually, and 20 randomly selected IdU-positive cells were then checked for Nestin positivity. In the ipsilateral SVZ, three sections per animal were evaluated. Here, two 300 μm x 300 μm counting windows were established, one was immediately adjacent to the corpus callosum, and one was located at the ventral border of the lateral ventricle. For each animal, 30 randomly selected IdU-positive cells were screened for Nestin co-expression.

*Analysis of Angiogenesis*

CD31 staining (angiogenesis staining) was quantitatively evaluated in the peri-infarct region using the ImageJ software program (version 1.48v). Three counting frames, each 596 μm x 447 μm in size, were positioned in the peri-infarct region. Two counting frames were positioned directly adjacent to the infarct area, and one was located immediately ventral to the infarct area at the level of the deepest infarct extent. These areas were photographed at 20x magnification. The CD31-positive structures were then traced on the photographs using the ImageJ program and their lengths calculated. The individual values of the vessel lengths per section were then set in relation to the examined area. The mean value of a brain and then of the respective test groups was determined by adding the values determined for the vessel lengths per area.

# Supplementary Figure Legends

**Supplemental Figure 1: Analysis of angiogenesis after photothrombotic stroke. (A)** Quantification of total vascular length per area, **(B)** Representative micrographs showing blood vessels (CD31) in the peri-infarct region. One-way ANOVA.


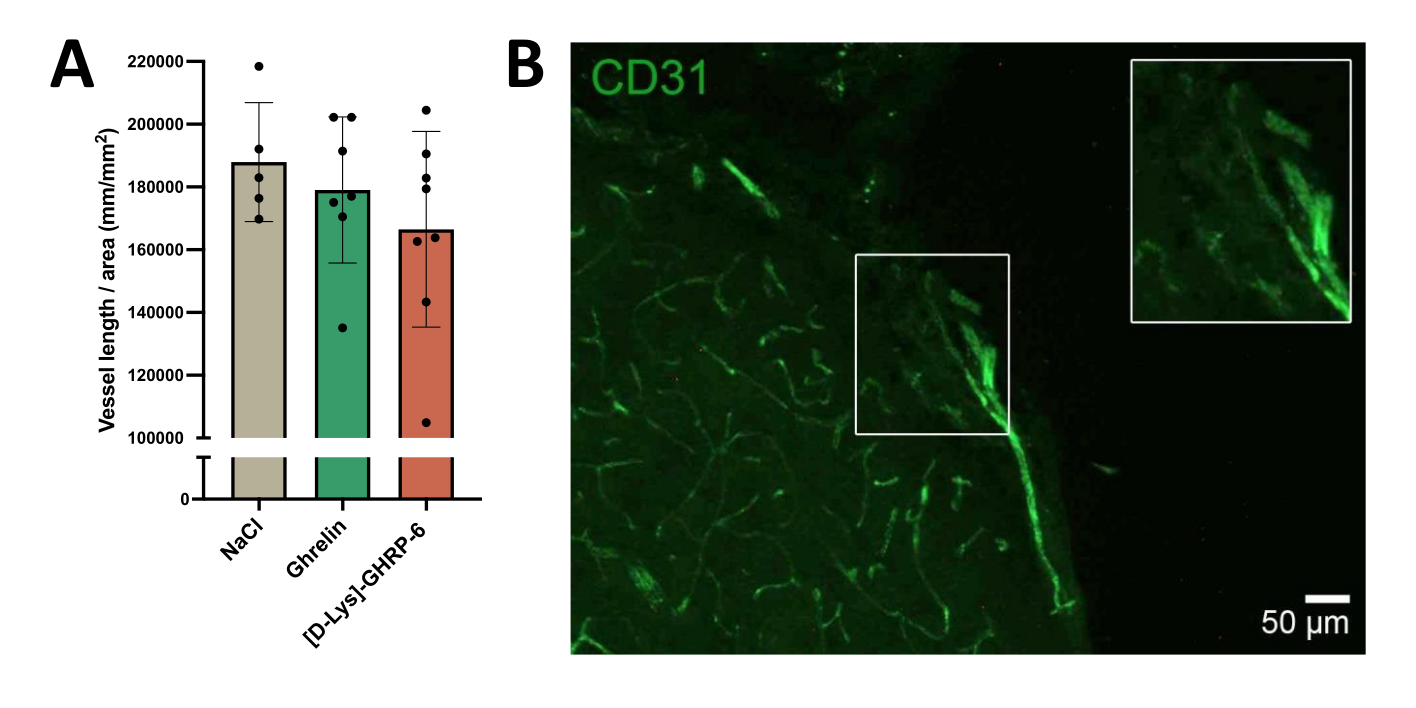


**Supplemental Figure 2: Attrition rate and number of subjects excluded from individual analysis and the rationale for exclusion for Experiment 1 (A) and Experiment 2 (B).**


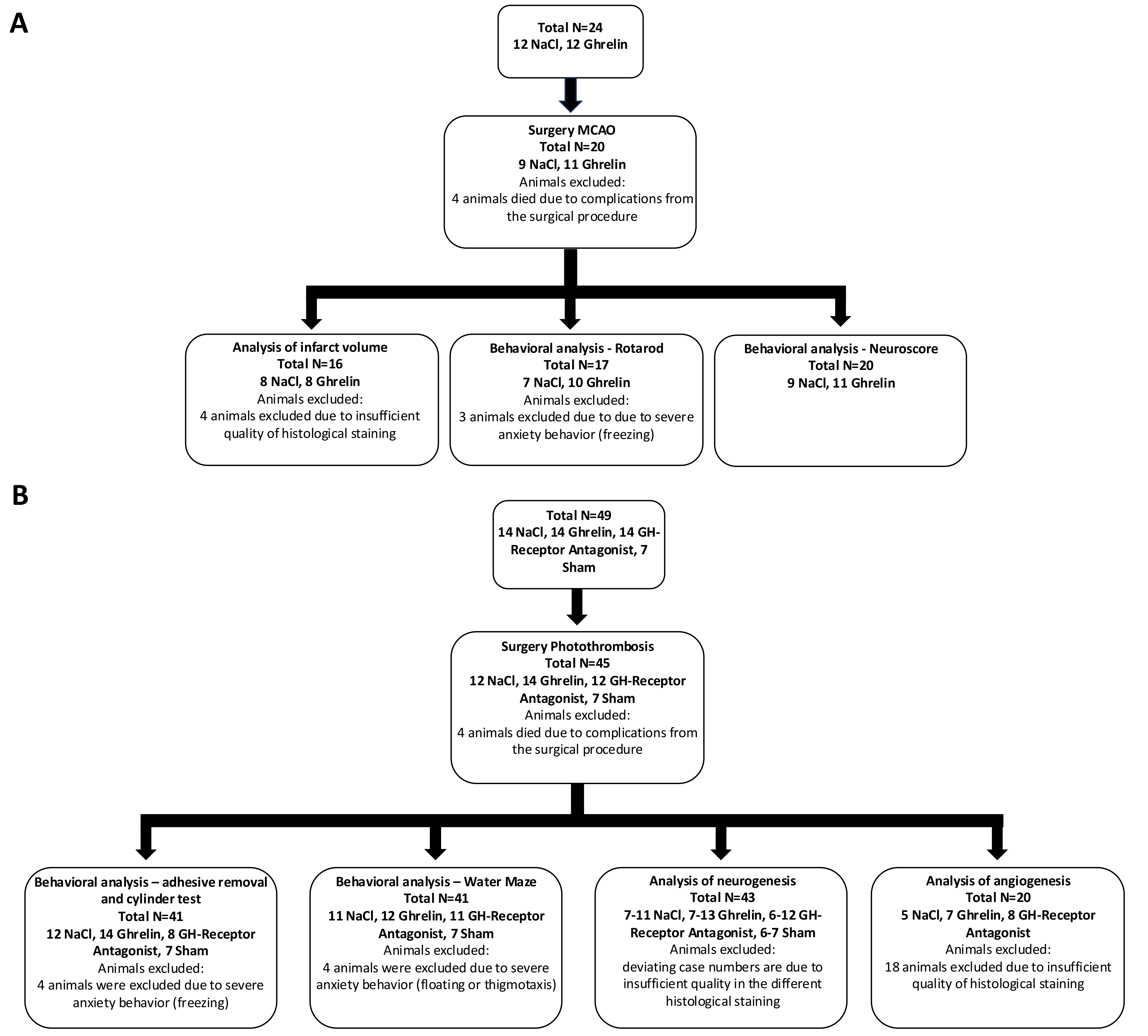

Supplement: Supplementary file 1 — Supplementary Material 1 [file 42466_2025_371_MOESM1_ESM.docx]
